# Mitral Valve Prolapse With Mitral Annular Disjunction Causing Ventricular Tachycardia During a Stress Test: A Case Report

**DOI:** 10.7759/cureus.64020

**Published:** 2024-07-07

**Authors:** Alfredo E Palomino Abella, Omar M Masarweh, Murali Iyyani, Volodymyr Oliynyk, Nicole Gill-Duncan

**Affiliations:** 1 Internal Medicine, University of Central Florida, Kissimmee, USA; 2 Cardiovascular Disease, Orlando Health, Orlando, USA; 3 Internal Medicine, Orlando VA Medical Center, Orlando, USA

**Keywords:** cardiac mri, cardiac stress test, ventricular tachycardia (vt), mitral valve prolapse, mitral annular disjunction

## Abstract

Mitral valve prolapse (MVP) is a relatively common valvular disorder characterized by displacement of one or both mitral valve leaflets into the left atrium (LA) during systole. Mitral annular disjunction (MAD) is an associated abnormality where a portion of the mitral valve annulus attaches superiorly in the left atrial wall. Although MVP is often considered benign, it can rarely lead to serious complications such as ventricular arrhythmias, especially when MAD is present. Herein, we present a case of a 63-year-old male with MVP and MAD who experienced sustained ventricular tachycardia (VT) during cardiac stress testing. This case underscores the importance of recognizing MVP with MAD as a potential substrate for ventricular arrhythmias, notably under heightened physiological or induced periods of stress.

## Introduction

Mitral valve prolapse (MVP) is a common valvular abnormality with an incidence of 0.6%-2.4% and is characterized by displacement of one or both mitral valve leaflets into the left atrium (LA) during systole [1]. It is often considered benign, but in some cases, it can lead to complications such as mitral regurgitation, infective endocarditis, arrhythmias, and sudden cardiac death [2]. Complicating this disorder may be concomitant mitral annular disjunction (MAD), which is a structural abnormality of the mitral annulus in which a portion of the mitral valve leaflet inserts into the atrial wall at a point distal from the junction of the left atrial and left ventricular (LV) myocardial tissue. This leads to a portion of the mitral annulus and its leaflets being inserted into the left atrium (LA) [3]. This abnormal displacement may lead to fibrosis in the region of the disjunction and may serve as a nidus for arrhythmias. The association between MVP with MAD and its predisposition to ventricular arrhythmias such as ventricular fibrillation (VF), ventricular tachycardia (VT), and premature ventricular contractions (PVCs) has been reported in multiple studies and case reports [4]. While MAD is increasingly recognized, its clinical significance, especially in association with MVP, remains an area of active investigation [1]. In this case, we report a patient who, during a nuclear stress test, experienced an episode of sustained ventricular tachycardia and was found to have MVP associated with MAD.

## Case presentation

**Figure 1:**
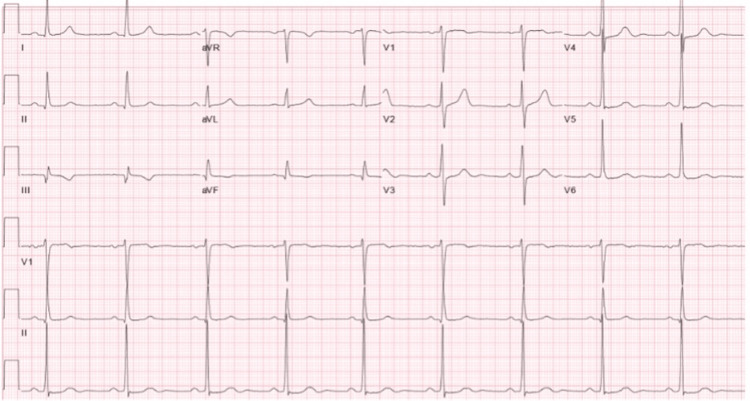
Baseline EKG prior to the stress test

**Figure 2:**
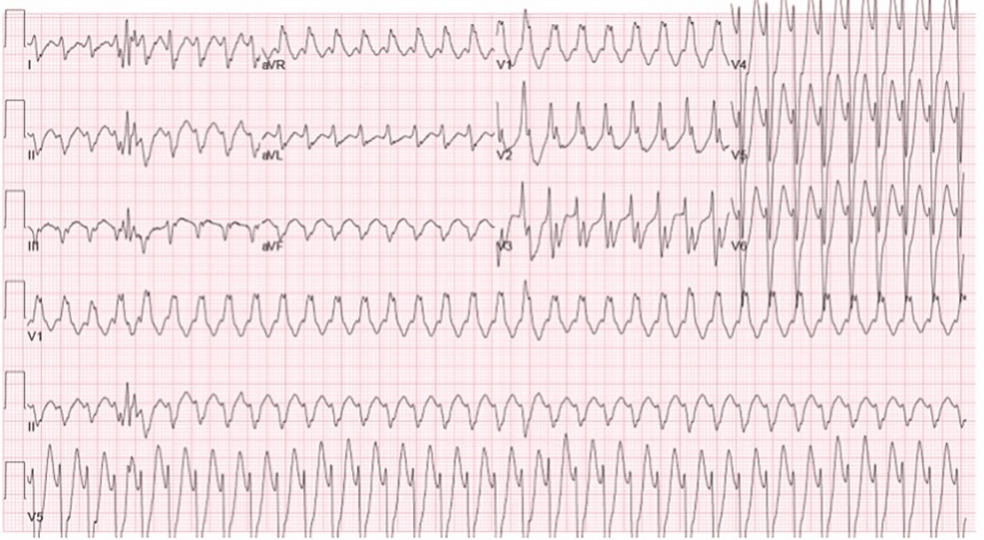
EKG during the stress test showing a wide complex tachycardia

A 63-year-old male with a history of hypertension and sickle cell trait was evaluated at an outpatient cardiology clinic following a recent overnight hospitalization at a community hospital due to lightheadedness and dizziness. He reported nonspecific chest pain lasting a few seconds, prompting a recommendation for an ischemic workup. While undergoing a nuclear stress test, the patient abruptly went into a wide complex tachycardia with a heart rate in the 200-205 bpm range (Figures 1, 2). He complained of palpitations with no associated chest pain or shortness of breath during this episode. His blood pressure remained stable throughout. A carotid massage and subsequent Valsalva maneuver were attempted by a supervising cardiologist without successful resolution of rhythm. At this time, a rapid response was called, where the patient was given 6 mg of intravenous adenosine, followed by 12 mg without a resolution of his arrhythmia. He was transferred to the emergency department where he was given 20 mg intravenous diltiazem bolus, followed by a repeat 25 mg intravenous diltiazem bolus, resulting in rate-controlled atrial fibrillation. He later spontaneously converted to sinus rhythm.

**Figure 3:**
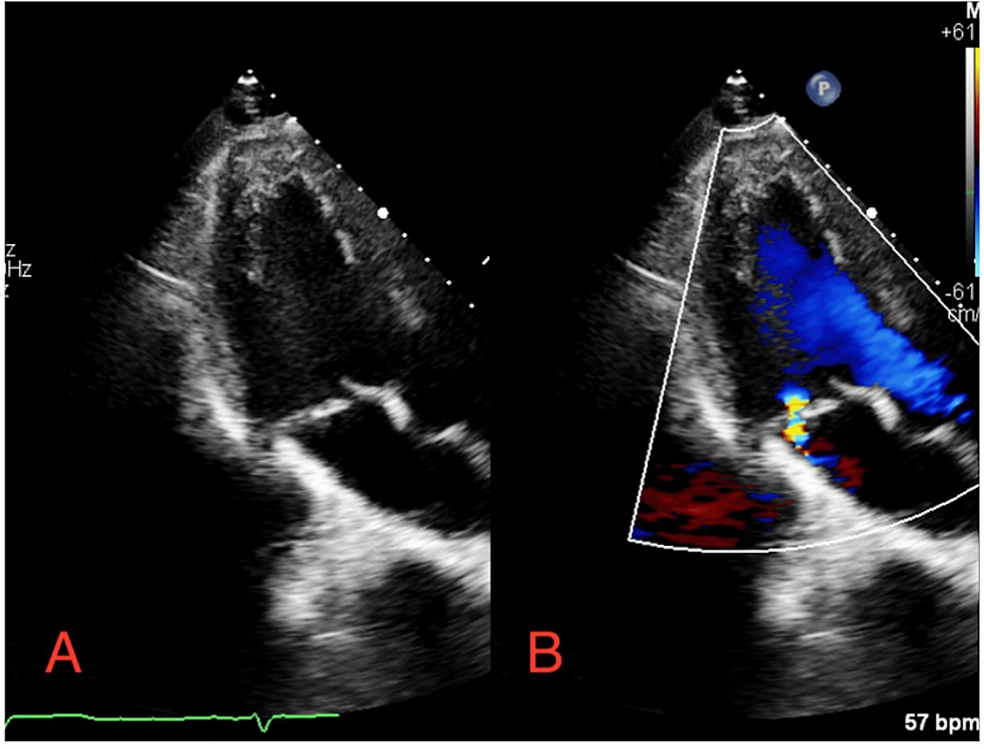
(A) Transthoracic echocardiogram in apical view showing notable MVP with a (B) posteriorly directed jet of mild mitral valve insufficiency with Doppler MVP: mitral valve prolapse

**Figure 4:**
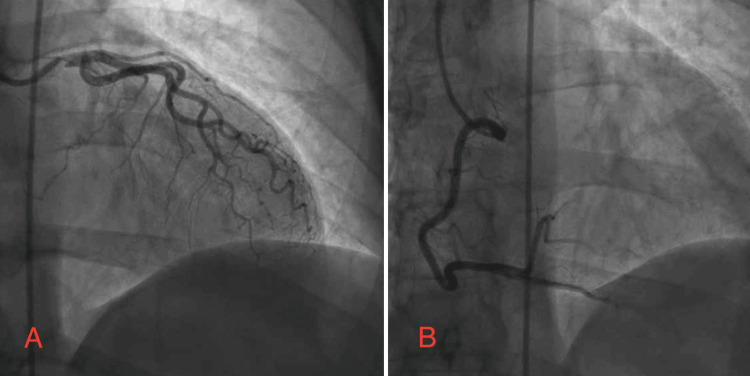
Angiography showing normal left ventricular end-diastolic pressure, no aortic stenosis, mild luminal irregularities, and no significant disease (A) No stenosis in the LM, LAD, or LCx. (B) No stenosis in the RCA. LM: left main, LAD: left anterior descending, LCx: left circumflex, RCA: right coronary artery

**Figure 5:**
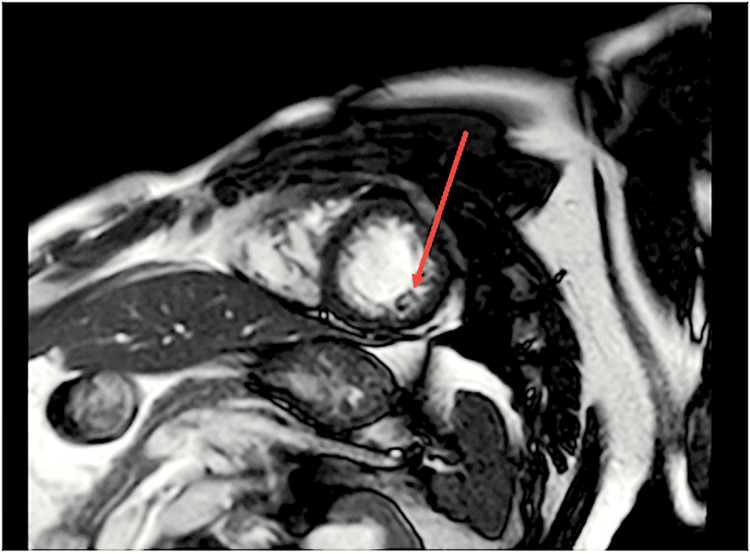
Normal LV/RV cavity size/thickness with LVEF of 58% and RVEF of 59% (arrow indicating the LV) LV: left ventricle, RV: right ventricle, LVEF: left ventricular ejection fraction, RVEF: right ventricular ejection fraction

**Figure 6:**
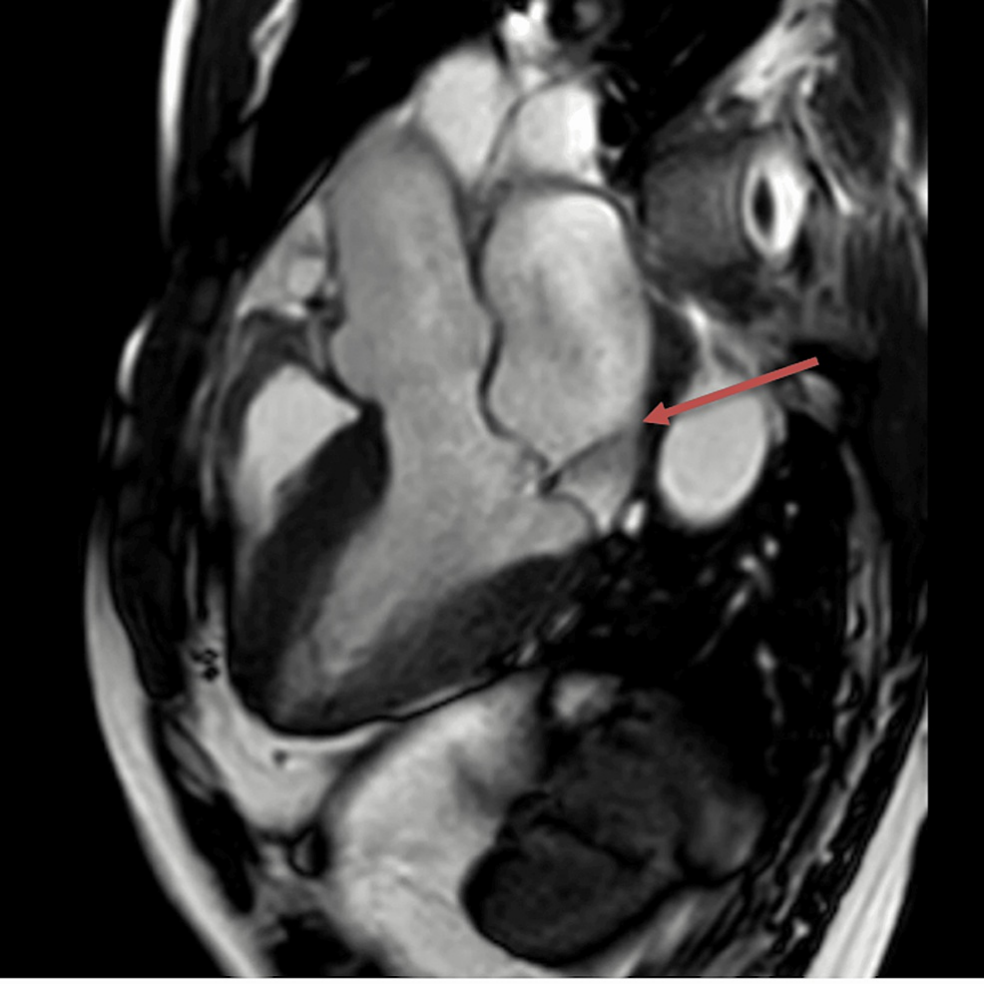
Cardiac MRI with an arrow indicating mild mitral valve regurgitation with posteriorly directed jet The regurgitant volume is 17 mL. The regurgitant fraction is 15%. MRI: magnetic resonance imaging

**Figure 7:**
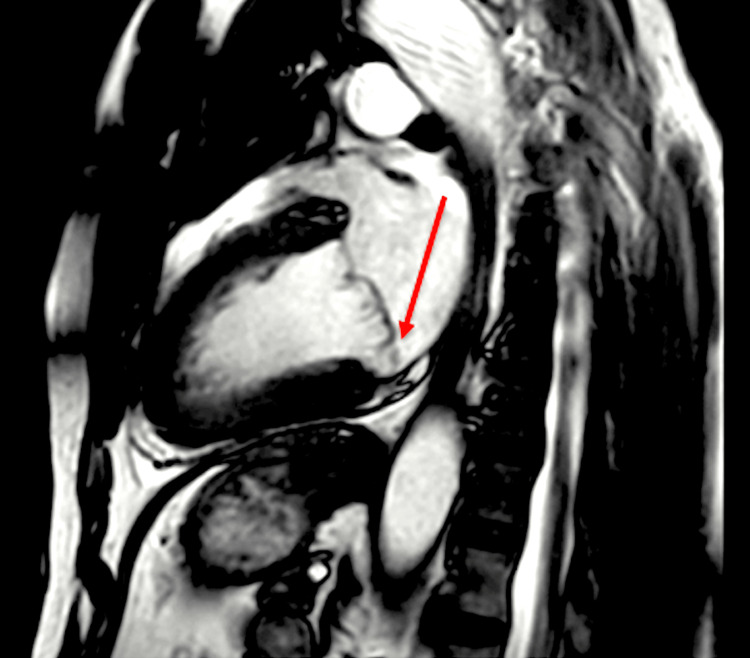
Cardiac MRI with an arrow indicating mild thickening and mild prolapse of the anterior mitral valve leaflet (predominantly A2 scallop) MRI: magnetic resonance imaging

**Figure 8:**
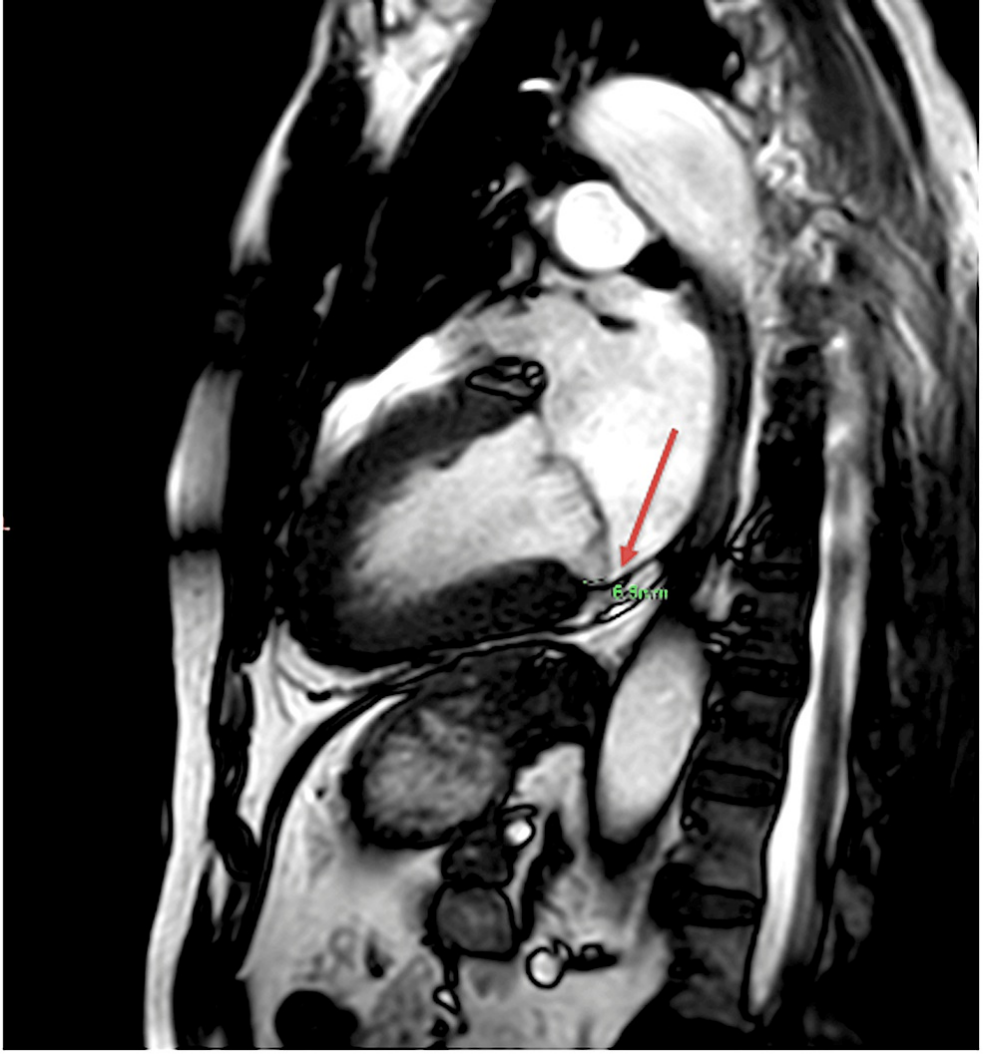
Cardiac MRI with an arrow indicating mild mitral annular disjunction (6.6 mm) MRI: magnetic resonance imaging

**Figure 9:**
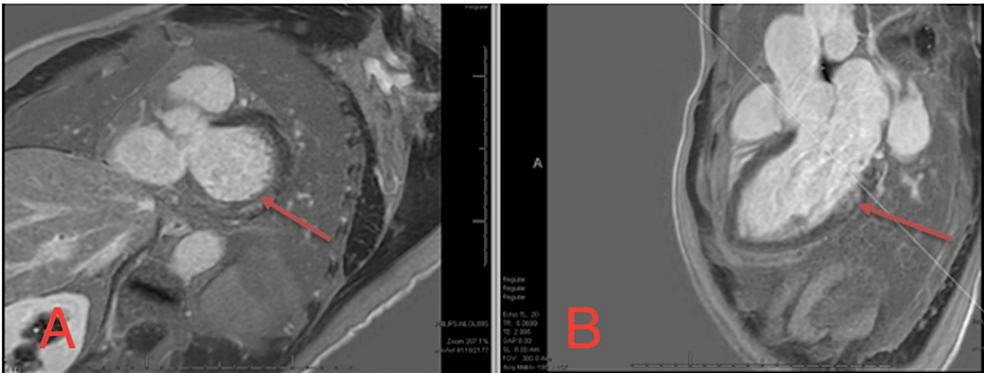
Mid-wall late gadolinium enhancement involving the base to the mid-inferolateral region as typically seen associated with mitral annular disjunction (arrow) (A) Short-axis view. (B) Long-axis view.

The patient was admitted to inpatient service for further workup, where he was monitored on telemetry and remained in sinus rhythm with no further events. An echocardiogram revealed a left ventricular (LV) ejection fraction of 45%-50% with mild global hypokinesis of the LV. There was notable MVP (possibly Barlow's valve) with a posteriorly directed jet of mild mitral valve insufficiency; no other abnormalities were seen (Figure 3). A left heart catheterization showed normal left ventricular end-diastolic pressure, no aortic stenosis with mild luminal irregularities, and no significant disease noted (Figure 4). Subsequent cardiac magnetic resonance imaging (CMR) findings revealed mild mitral annular disjunction associated with mild thickening and prolapse of the anterior mitral valve leaflet (Figures 5-9). The patient underwent evaluation by the electrophysiology team due to his sustained ventricular tachycardia and received an automatic implantable cardioverter (AICD). Additionally, he was started on a long-acting calcium channel blocker, and his home losartan was resumed. He was discharged with outpatient cardiology follow-up.

## Discussion

Mitral valve prolapse has been defined as a superior displacement of the mitral leaflets above the mitral annulus greater than 2 mm during systole as measured by an echocardiogram [5,6]. Anatomically, the mitral annulus comprises the anterior and posterior leaflets with the anterior leaflet being part of the fibrous trigone and the aortic annulus, making it less prone to disjunction. Conversely, the posterior leaflet, comprising two-thirds of the annulus, is much thinner and attaches to the ventricular myocardium and the fibrous end of the left atrial wall. Therefore, the mitral annulus provides support for the leaflets and serves to isolate electrical activity between the atria and the ventricle. This relatively thin fibrous connection along the posterior leaflet allows disjunction to occur [1]. MVP and its association with ventricular arrhythmias has garnered increasing attention in recent years, particularly in the context of concomitant mitral annular disjunction. MAD, broadly defined as a separation of the left atrial wall at the point of mitral valve insertion and the left ventricular free wall, has been associated with degeneration of the mitral valve as well as arrhythmias and sudden cardiac death (SCD) [3]. While the association between MVP and MAD has been confirmed, there does not appear to be a correlation between MVP severity and MAD [3].

Histologically, MAD causes stretching of the mitral annulus, and the typical atrial tissue that would be present is often replaced by fibrous tissue, which is thought to be the source of the associated arrhythmias [3]. Grossly, MAD is best observed in late systole on imaging studies where there is a separation between the attachment of the posterior mitral valve leaflet to the LA wall and the LA-LV junction. It can be defined as the absence of myocardium during systole between the mitral valve annulus and the adjacent segment of the left ventricular wall. The distance of separation is often termed the MAD length or distance, with most studies using a cutoff of at least 2 mm. However, different cutoff lengths have been used in multiple studies depending on the imaging technique used, ranging from 1 mm to 5 mm [3]. Transthoracic echocardiogram (TTE) in the parasternal long-axis view has a reported sensitivity of 65% and specificity of 95%; however, cardiac magnetic resonance imaging (CMR) appears to be more sensitive and has higher detection rates [3]. Furthermore, CMR can provide additional information such as evaluating myocardial function, providing better spatial resolution than an echocardiogram, and detecting scar tissue [7].

The pathogenesis of ventricular arrhythmias in MVP with MAD is multifactorial and has not been fully elucidated. However, several mechanisms have been proposed. Firstly, the abnormal mechanical stress on the ventricular myocardium caused by the displacement of mitral valve leaflets in MVP, compounded by the separation of the mitral annulus from the left ventricular myocardium in MAD, may lead to myocardial fibrosis and electrical instability [8]. This fibrosis creates a substrate for reentrant circuits, facilitating the initiation and maintenance of ventricular arrhythmias. Fibrosis, irrespective of MVP or MAD, when noted on CMR as having late gadolinium enhancement, has repeatedly been shown to be associated with ventricular arrhythmias and sudden cardiac death [3]. Secondly, the presence of MVP and MAD may result in alterations in ventricular repolarization and dispersion of refractoriness, predisposing individuals to ventricular ectopy and arrhythmias. Additionally, the abnormal stretch on the papillary muscles and chordae tendineae in MVP can lead to mechanical triggers for arrhythmias, such as premature ventricular contractions (PVCs) or ventricular tachycardia [9]. Furthermore, autonomic dysfunction, which is common in patients with MVP, may contribute to arrhythmogenesis by modulating cardiac electrophysiology and promoting arrhythmic triggers, particularly during periods of sympathetic activation such as exercise or cardiac stress testing, as in the case of our patient [1].

The occurrence of VT during stress testing in patients with MVP and MAD poses significant clinical implications. Firstly, it underscores the need for comprehensive evaluation and risk stratification in individuals with these conditions, even in the absence of significant structural heart disease. While MVP has traditionally been considered a benign entity, the presence of MAD and associated ventricular arrhythmias may confer a higher risk of adverse cardiac events, including sudden cardiac death. Moreover, the identification of VT during stress testing may serve as a marker of disease severity and progression in patients with MVP and MAD. It highlights the importance of vigilant monitoring and timely intervention to mitigate the risk of sudden cardiac death in this population.

Management strategies for patients with MVP and MAD presenting with VT during stress testing encompass both acute and long-term approaches. Acutely, prompt termination of the arrhythmia with administration of antiarrhythmic medications may be necessary to restore sinus rhythm and stabilize hemodynamics. Long-term management involves risk stratification for sudden cardiac death and consideration of implantable cardioverter-defibrillator (ICD) placement in high-risk individuals. The decision to implant an ICD should be individualized based on the presence of additional risk factors, such as a history of syncope, sustained VT, or significant ventricular ectopy burden on ambulatory monitoring. Currently, there is no role of ICD placement for primary prevention in these cases; however, ICD placement for secondary prevention is generally indicated [2]. Other options such as catheter ablation have been studied. Syed et al. [10] demonstrated that catheter ablation of ventricular ectopic foci in patients with bileaflet MVP is effective in reducing symptomatic ventricular ectopy and reduced the number of ICD shocks in patients with previous cardiac arrest; however, no guidelines for the role of catheter ablation in such cases has been delineated.

Further research is warranted to enhance our understanding of the pathophysiology of ventricular arrhythmias in MVP with MAD and to refine risk stratification and treatment algorithms for affected individuals. Prospective studies investigating the natural history of arrhythmias in this population, associated risk factors, and the efficacy of various therapeutic interventions are needed to inform evidence-based management strategies and improve clinical outcomes.

## Conclusions

In conclusion, sustained ventricular tachycardia during stress testing in patients with MVP and MAD represents a potentially life-threatening complication that requires vigilant clinical assessment and management. A multidisciplinary approach involving cardiologists, electrophysiologists, and imaging specialists is essential to optimize risk stratification, treatment, and long-term follow-up in this high-risk population.
